# Gastric diverticula as a diagnostic and therapeutic challenge: Case report and review of literature

**DOI:** 10.1016/j.ijscr.2020.04.053

**Published:** 2020-05-11

**Authors:** Imen Akkari, Mohamed Hedi Mraiedha, Imen Jemni, Soumaya Mrabet, Fehmi Hamila, Ilhem Ben Jazia, Rached Letaief

**Affiliations:** a Department of Gastroenterology, Farhat Hached Hospital, Faculty of Medicine of Sousse, University of Sousse, 4000, Sousse, Tunisia; b Department of Digestive Surgery, Farhat Hached Hospital, Faculty of Medicine of Sousse, University of Sousse, 4000, Sousse, Tunisia

**Keywords:** Gastric diverticulectomy, Laparoscopic gastric surgery, Diverticula, Dyspepsia

## Abstract

**Introduction:**

Gastric diverticula are a rare condition characterized by a pouch protruding from the gastric wall. It is commonly asymptomatic and managed without surgery. Gastric diverticulotomy is indicated in the case of symptomatic or complicated diverticula.

**Case:**

The authors report the case of a 67 year-old man presenting with heartburn and dyspepsia with no response to proton pump inhibitors. The patient was diagnosed with a gastric diverticula and a little hiatal hernia, which was subsequently successfully treated with a laparoscopic gastric diverticulectomy and Nissen fundoplication.

**Conclusion:**

Gastric Diverticula have to be evoked in the absence of response of Gastro Esophageal Reflux Disease to therapy or in atypical symptoms. Laparoscopic resection of GD can be safe with resolution of symptoms.

## Highlights

• Gastric Diverticula is a rare disease.
• The long history and the non specificity of symptoms may sometimes lead to a misdiagnosed disease.
• Diagnosis is based on Video endoscopic and Upper gastrointestinal contrast radiographic study.
• Surgical resection is recommended when symptoms persist despite adequate medical therapy.
• Laparoscopic resection is considered a safe and suitable procedure.

## 1 Introduction

Gastric diverticula (GD) are a rare entity of the gastrointestinal tract. It is accidentally identified or during endoscopic or radiological exploration for superior digestive tract symptoms. The prevalence is 0.04% on gastric radiographic examinations and 0.01 to 0.11% at endoscopy [1,2].

The majority of GD cases are asymptomatic. However, occasionally abdominal symptoms occur, ranging from dyspepsia to major upper gastrointestinal bleeding or perforation [2,3].

The diagnosis is based on endoscopic and radiologic explorations [2].

The treatment is indicated in case of symptomatic GD. It depends on the severity of symptoms, the size of diverticulum and the presence of complications [2,4].

The aim of this report is to describe a rare symptomatic gastric diverticulum and its laparoscopic therapeutic challenges. A litterature review was also performed to investigate such management.

## 2 Case report

A 67 year-old woman with no past medical or surgical history, was presented with persistent dyspepsia and heart burn without respond to proton pump inhibitor. The patient denied weight loss, hematemesis or other symptoms. Physical examination was negative. The laboratory investigations were normal.

**Fig. 1:**
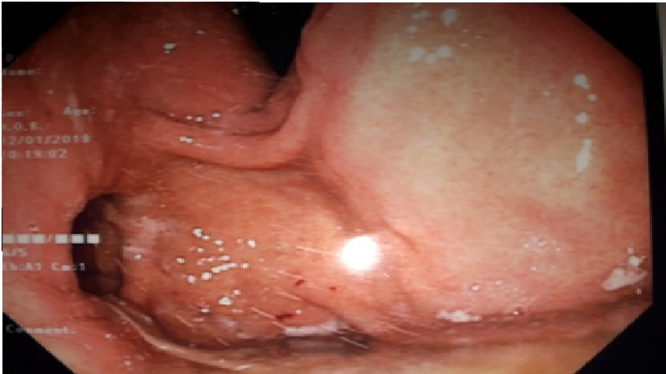
Upper gastrointestinal endoscopy: A subcardial diverticula directed posteriorly off the fundus of the stomach.

**Fig. 2:**
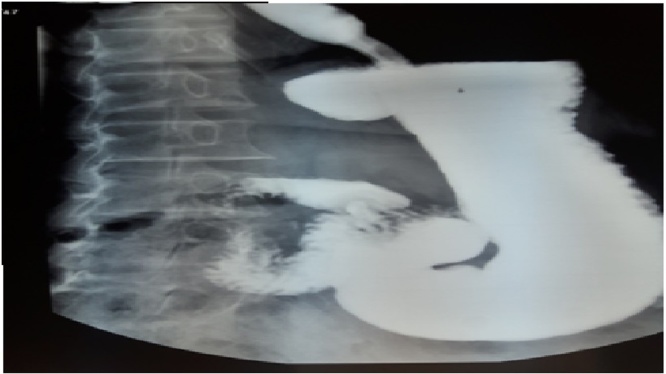
Upper gastrointestinal contrast image of the gastric diverticulum.

His symptoms were suggestive of a gastro esophageal reflux (GERD). Upper video endoscopy shows a subcardial diverticula directed posteriorly off the fundus of the stomach. It was approximately 3 cm in diameter, (Fig. 1) with hiatal hernia and without œsophagitis. High resolution esophageal manometry revealed a normotonic sphincter which relaxes well in 100% of swallowing and absence of esophageal contractility in 100% of swallowing. The esophago-gastric barium study had showed a protruding pouch in the upper gastric region (Fig. 2). The Abdominal computerized tomography (CT) with intravenous (IV) contrast media and negative oral contrast media (water) had showed normal stomach without any evidence of diverticulum.

**Fig. 3:**
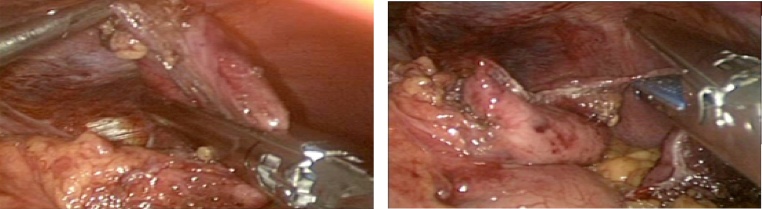
Laparoscopic resection of gastric diverticula.

The operation was performed laparoscopically. It had revealed a 3 cm hiatal hernia, than after further dissection of the hiatus and upper part of the stomach, a 3 cm diverticulum on the posterior wall of the fundus. A laparoscopic stapler (EndoGIA* covidien), resection of the diverticulum was performed (Fig. 3) followed by a Floppy Nissen fundoplication.

The patient was discharged home on day 1 after surgery. At 3 month follow-up, the patient has no reflux symptoms and no dysphagia.

## 3 Discussion

Gastric Diverticula is a rare disease that usually present in the fifth and sixth decades of life, with no sex predominance [2,5].

GD can be congenital or acquired. Congenital type, also called true diverticula, constitutes 75% of GDs [6]. They are most commonly found on the posterior wall of the stomach and near the gastro esophageal junction. Therefore our case seems to be congenital. The acquired GD are a pseudodiverticula, usually located in the antrum and associated with other gastrointestinal pathologies [2,6,7].

The range of diameter is commonly 1–3 cm [1]. It seems that clinical presentation depends on the diverticula size [1]. Symptoms, complications and resistance to medical therapy are more frequent in the case of a diverticula larger than 4 cm [6]. Atypical symptoms, GERD with no response to proton pump inhibitors must evoke GD. This proximal gastric pouch represents a secondary tank, seat of acid and food stasis that can be a cause of regurgitation.

Vague upper abdominal and epigastric pain are the most common symptoms, encountered in 18%–30% of cases of symptomatic patients. Other symptoms may be observed ranging from vomiting, anorexia, dysphagia, Food retention, halitosis…. [4,5] to severe complications such as perforation or hemorrhagic shock, torsion and malignancy [2,8,9].

The long history and the non specificity of symptoms may sometimes lead to a misdiagnosed disease. According to Palmer [10], in 30 of 49 symptomatic patients with a GD, symptoms were attributable to other gastrointestinal diseases.

Video endoscopic and Upper gastrointestinal contrast radiographic study are required for the GD diagnosis [11,12].

Although the performance of these methods for detecting GD they can still miss the lesion if it has a narrow neck that precludes entry of the contrast or scope, giving false negative results [3]. In addition, Oesophagogastrodudenoscopy is operator dependant and can misdiagnosed a small diverticulum less than < 2 cm [8].

To improve the sensibility of radiologic exam evaluation, it is recommended to use a right, anterior oblique view with the patient in a supine, slightly left lateral decubitus and Trendelenburg position [2].

There is no indication for treatment in the case of asymptomatic GD [2]. Medical treatment, such as protein pump inhibitors, antacids, or antispasmodics, have all been reported to relieve symptoms [13].

Surgical resection is recommended when symptoms persist despite adequate medical therapy, but also when the GD is large (more than 4 cm), bleeding, infected, perforated or the seat of a tumor [4,11,13]. Laparoscopic resection is considered a safe and suitable procedure [4,10,14].

A complete resolution after diverticulectomy has been noted in the case of halitosis [13]. In presence of persistent gastro esophageal reflux symptoms, complaints was managed using proton pump inhibitors in one case [15]. In another one, a laparoscopic Nissen fundoplication with simultaneous diverticulotomy was performed with resolution of symptoms [1].

In our case, the persistence of dyspepsia and reflux symptoms despite medical therapy was considered as indication for surgical management. On the way of Gockel et al. laparoscopic Nissen fundoplication with simultaneous diverticulectomy was performed with resolution of symptoms

## 4 Conclusion

GD has to be evoked in the absence of response of GERD to therapy or in atypical symptoms. Laparoscopic resection of GD can be safe with resolution of symptoms.

This work has been reported in line with the SCARE criteria according to SCARE guidelines [16].

## Declaration of Competing Interest

No conflicts of interest.

## Funding

NA.

## Ethical approval

Study is exempt from ethnical approval in our institution.

## Consent

We have obtained the consent from the patient to publish this case.

## Author contribution

Elhem Ben Jazia: study concept.

Imen Akkari, imen jemni: writing the paper.

Rached Ltaief, Fehmi Hmila: data collection.

Mohamed hédi mraidha, soumaya mrabet: data analysis.

All authors read and approved the final manuscript.

## Registration of research studies

NA.

## Guarantor

Imen Akkari.

## Provenance and peer review

Not commissioned, externally peer-reviewed.

## References

[1] Gockel I., Thomschke D., Lorenz D. (2008). Gastrointestinal: gastric diverticula. J. Gastroenterol. Hepatol..

[2] Rashid F., Aber A., Iftikhar S.Y. (2012). A review on gastric diverticulum. World J. Emerg. Surg. WJES.

[3] Marano L., Reda G., Porfidia R., Grassia M., Petrillo M., Esposito G. (2013). Large symptomatic gastric diverticula: two case reports and a brief review of literature. World J. Gastroenterol..

[4] Muis M.O., Leitao K., Havnen J., Glomsaker T.B., Søreide J.A. (2014). Gastric diverticulum and halitosis—a case for surgery?. Int. J. Surg. Case Rep..

[5] DuBois B., Powell B., Voeller G. (2012). Gastric diverticulum: “a wayside house of ill fame” with a laparoscopic solution. JSLS.

[6] Mahafza W.S., Taib A.A., Shahait A.D., Al Awamleh A. (2015). Chronic gastritis in a gastric diverticulum misdiagnosed as a left adrenal mass. Indian J. Surg..

[7] MaCauley M., Bollard E. (2010). Gastric diverticulum: a rare cause of refractory epigastric pain. Am. J. Med..

[8] Perbet S., Constantin J.-M.-M., Poincloux L., Privat J., Bannier F., Cayot-Constantin S. (2008). Gastric diverticulum: a rare cause of hemorrhagic shock. Intensive Care Med..

[9] Sasani H., Ekmen N., Sasani M. (2016). The importance of imaging in the diagnosis of gastric diverticulum: a case report. Int. J. Anat. Var..

[10] Palmer E. (1951). Gastric diverticula. Int. Abstr. Surg..

[11] Feng Y.E., Zhang Z. (2015). Gastric diverticulum simulating a left adrenal mass: a case report and review of the literature. Oncol. Lett..

[12] Araki A., Shinohara M., Yamakawa J., Tanaka M., Natsui S., Izumi Y. (2006). Gastric diverticulum preoperatively diagnosed as one of two left adrenal adenomas. Int. J. Urol..

[13] Zelisko A., Rodriguez J., El-Hayek K., Kroh M. (2014). Laparoscopic resection of symptomatic gastric diverticula. JSLS.

[14] Globke B., Fikatas P., Beck A., Adler A., Schmidt S.C. (2013). Multidisciplinary laparo-endoscopic management of a subcardial gastric diverticulum. Endoscopy.

[15] Eitzen K., Eslick G.D., Daneshjoo R. (2012). Dyspepsia and gastroesophageal reflux symptoms predominate in gastric diverticulum. J. Dig. Dis..

[16] Agha R.A., Borrelli M.R., Farwana R., Koshy K., Fowler A., Orgill D.P., For the SCARE Group (2018). The SCARE 2018 statement: updating consensus surgical CAse REport (SCARE) guidelines. Int. J. Surg..

